# Optimization of β-Carotene Enrichment of Coconut Oil from Canistel (*Pouteria campechiana* L.) Using Response Surface Methodology

**DOI:** 10.3390/foods14223947

**Published:** 2025-11-18

**Authors:** Harshaka Maduwantha Jans, Madushi Chathurika Wijerathna, Ganwarige Sumali Nivanthi Fernando, Maryam S. Hafiz

**Affiliations:** 1 Department of Food Science and Technology, Faculty of Agriculture, University of Ruhuna, Mapalana, Kaburupitiya, Matara 81000, Sri Lanka; harshakalogon@gmail.com (H.M.J.); chathurikamadushi123@gmail.com (M.C.W.); 2 Department of Clinical Nutrition, Faculty of Applied Medical Sciences, King Abdulaziz University, Jeddah 21589, Saudi Arabia

**Keywords:** β-carotene, *Pouteria campechiana*, coconut oil, antioxidant activity, response surface methodology (RSM)

## Abstract

Vitamin A deficiency (VAD) affects millions of people around the world, particularly in populations with limited dietary diversity. The present study aimed to develop β-carotene-fortified coconut oil using *Pouteria campechiana* (Canistel), which is an underutilized tropical fruit rich in β-carotene. A solvent-free fortification process was optimized using response surface methodology (RSM) with a central composite design to evaluate the effects of oil percentage (50–100% *w*/*v*) and heating temperature (30–60 °C) on β-carotene retention and oil quality. The quadratic model showed excellent fit (R^2^ = 0.9970; lack of fit *p* = 0.6815), while optimum conditions were identified at 52.4 °C and 50% (*w*/*w*) oil, yielding a predicted β-carotene concentration of 2.22 µg/mL. The fortified oil exhibited significantly higher polyphenolic (17.37 ± 2.04 mg GAE/g), total flavonoid (11.33 ± 0.89 mg QE/g) contents, and antioxidant activity (9.78 ± 0.53 mg TE/g) compared with commercial coconut oil. The optimized oil demonstrated enhanced oxidative stability, reflected by lower peroxide (0.05 ± 0.01 meqO_2_/kg) and acid values (0.10 ± 0.01 mg KOH/g). The validated quadratic model and optimized process produced a nutritionally enriched, stable coconut oil, supporting its use as a clean-label functional ingredient. These findings highlight the potential of β-carotene-fortified oils to deliver provitamin A and antioxidant benefits. Future studies should focus on evaluating the bioavailability, storage stability, and sensory acceptability of β-carotene enriched coconut oil to confirm its nutritional and commercial potential.

## 1. Introduction

Vitamin A deficiency (VAD) remains a major global public health concern, particularly in developing regions where limited dietary diversity and insufficient supplementation exacerbate micronutrient malnutrition [1]. VAD is associated with severe visual impairment, including night blindness and xerophthalmia, and increases the risk of infections, impaired immune responses, and maternal and child mortality [2]. It is a sustainable strategy for improving public health outcomes to address this deficiency through safe, diet-based approaches.

Among the natural provitamin A sources, β-carotene is the most abundant and physiologically significant carotenoid, serving as a precursor to retinol (vitamin A). In the human body, β-carotene is enzymatically cleaved in the intestinal mucosa by β-carotene-15,15′-dioxygenase to produce retinal, which is subsequently reduced to retinol, the bioactive form of vitamin A [3]. Major dietary sources of β-carotene include carrots, sweet potatoes, pumpkins, mangoes, papayas, and leafy vegetables such as spinach and kale. However, the availability and consumption of these foods are often limited in low-income populations.

Recent trends in functional food research have focused on fortifying commonly consumed food matrices with natural antioxidants and bioactive compounds to enhance micronutrient intake [4,5]. Coconut oil (*Cocos nucifera*) is a widely used edible lipid in tropical regions, known for its high oxidative stability, medium-chain triglyceride profile, and excellent capacity to solubilize lipophilic compounds like β-carotene [6]. Coconut oil was selected as the extraction medium because of its high proportion of medium-chain saturated fatty acids, which enhance solubilization of lipophilic carotenoids and provide good oxidative stability. In addition, coconut oil is locally abundant and widely accepted in Sri Lankan food systems. The broad application of coconut oil in the food, cosmetic, and pharmaceutical sectors makes it an ideal carrier for developing fortified functional oils.

*Pouteria campechiana* (Canistel or eggfruit) is an underutilized tropical fruit native to Central and South America and parts of Asia, including Sri Lanka. The fruit contains bright yellow-orange, creamy pulp rich in carotenoids, particularly β-carotene, as well as other bioactive compounds such as polyphenols, flavonoids, and vitamins B and C [7,8]. The carotenoid profile is dominated by neoxanthin and violaxanthin, followed by β-carotene, β-cryptoxanthin, and zeaxanthin, which collectively contribute to the fruit’s deep yellow coloration and antioxidant activity. The presence of phenolic acids (gallic, ferulic, caffeic) and flavonoids (catechin, epicatechin, quercetin, and myricetin) further enhances its functional potential [8]. This composition indicates that *P. campechiana* is a valuable natural source of both lipophilic and hydrophilic antioxidants suitable for oil-based fortification applications. Despite its high nutritional value, Canistel remains largely underexploited due to limited consumer awareness, short shelf-life, and a lack of processing and value addition strategies. Although it is widely grown in tropical and sub-tropical regions, its potential to reduce micronutrient deficiency is unexploited [9]. The use of Canistel as a source of vitamin A is a promising approach to enhancing dietary intake of provitamin A, as well as supporting local agrobiodiversity, minimizing postharvest losses, and promoting functional food development. In addition, the incorporation of bioactive-rich pulp in commonly consumed food matrices, such as edible oils, aligns with a current trend in functional food research aimed at enhancing micronutrient intake and public health outcomes in foods that contain natural antioxidants [4,5].

Despite the nutritional potential of *Pouteria campechiana* (Canistel) as a β-carotene source, limited studies have explored its incorporation into edible oils, particularly under solvent-free conditions [10,11]. Most of the existing research emphasizes solvent-based extraction methods, which may not align with the growing clean-label products demand [12]. Furthermore, critical processing parameters such as heating temperature and oil-to-pulp ratio remain unoptimized, potentially affecting carotenoid retention and oil quality. Optimizing these parameters is essential to enhance the stability and bio accessibility of β-carotene, ensuring its efficacy in addressing vitamin A deficiency. Recent studies have highlighted the importance of process optimization in carotenoid extraction, emphasizing the need for systematic approaches to improve yield and stability [12]. Oil-based extraction not only offers a solvent-free, food-grade approach but also enhances carotenoid solubility and protects these thermolabile pigments from oxidative degradation. Incorporating carotenoids into edible oils improves their bioavailability, as lipid micelles facilitate intestinal absorption during digestion [6,12]. Therefore, coconut oil serves both as an extraction medium and a carrier system for improved nutritional delivery.

Therefore, this study aimed to develop β-carotene-fortified coconut oil using *Pouteria campechiana* through a green, solvent-free approach. Response Surface Methodology (RSM) was employed to optimize key process parameters for maximal β-carotene retention and antioxidant activity. The fortified oil was evaluated for its nutritional, physicochemical, and functional properties in comparison with commercial coconut oil. The findings highlight the potential of Canistel-based β-carotene enrichment as a sustainable strategy to develop clean-label functional oils that support nutritional security, food fortification and value addition to underutilized crops.

## 2. Materials and Methods

### 2.1. Materials and Sample Preparation

The fruits of *Pouteria campechiana* were collected from Kandy, central province (7.183610° N, 80.585109° E) in Sri Lanka and stored in deep-frozen conditions at −20 °C in a deep freezer (Singer chest freezer, Singer, Sri Lanka) throughout the research study. Commercially available refined coconut oil from a local market in Kandy, Sri Lanka, was used as a reference. The pulp of Pouteria was mechanically separated from the peel and seeds using a sterile spatula, and the resulting material was collected at ambient laboratory temperature (28 ± 2 °C) for subsequent processing. All reagents used were of analytical grade.

### 2.2. Experimental Design for Optimization of β-Carotene Enrichment

A response surface methodology (RSM) with central composition design (CCD) was used to optimize the β-carotene enrichment process of coconut oil. Two independent variables, such as heating temperature of oil (X_1_, °C) and oil-to-solid ratio (X_2_, % *w*/*w*) were selected and evaluated for their effect on β-carotene retention (Y) (Table 1). These factors were chosen based on preliminary trials and supporting literature indicating their dominant influence on carotenoid transfer efficiency during solvent-free oil enrichment, while other variables such as extraction time and agitation speed were maintained at a constant level to ensure process simplicity and reproducibility. The CCD contained 13 runs, including five central points and four axial points arranged in a randomized order to minimize systematic bias. The experimental data were fitted to a second-order polynomial model to evaluate the linear, quadratic, and interaction effects of the variables on β-carotene retention as shown below in Equation (1).

Y = βo + β_1_X_1_ + β_2_X_2_ + β_1_β_2_X_1_X_2_ + β_1_^2^X_1_^2^ + β_2_^2^X_2_^2^ (1)

where Y represents the predicted response (β-carotene content, µg/mL). βo is the intercept, β_1_, β_2_ are linear coefficients, β_1_β_2_ represents the interaction effect and β_1_^2^ and β_2_^2^ are the quadratic coefficients.

The factor levels were coded as −1 (low), 0 (center), and +1 (high) according to the following equation:

Z = (Zo − Zc)/∆Z(2)

where Z is the coded variable, Zo indicates the actual value, Zc the value at the central point, and ∆Z represents the step change between levels.

The regression coefficients were determined, and 3D surface and contour plots were generated to visualize the effects of factors and identify the optimum enrichment conditions.

#### 2.2.1. Process of β-Carotene Enrichment

According to the suggested treatment combinations generated by the RSM design, the required quantities of *Pouteria campechiana* pulp and coconut oil were mixed and blended for 15 min at room temperature (28 ± 2 °C). The mixture was then filtered through a muslin cloth to separate the oil phase, and this extraction procedure was repeated five times to ensure maximum recovery of β-carotene. The filtrate was centrifuged at 1500× *g* for 10 min, and the supernatant (β-carotene-enriched coconut oil) was collected for further analysis and stored at −20 °C in a capped glass test tube, covered with aluminum foil.

#### 2.2.2. Process of Heating to Determine the Retention of β-Carotene in Enriched Coconut Oil

For each of the 13 treatment combinations, 50 mL of the samples were accurately measured and transferred into 100 mL beaker. The sample was subjected into controlled heating on a magnetic stirrer hot plate (AREX 5 Digital, VELP Scientifica, Usmate, Italy), with continuous agitation to ensure uniform temperature distribution. The temperature of each sample was monitored in real time using a calibrated thermometer, and heating continued until the predetermined target temperature was achieved.

### 2.3. Sample Preparation for β-Carotene Determination

Extraction of β-carotene present in the enrich oil was carried out according to the method described in [13] with minor modifications. One milliliter of oil samples from each treatment was mixed with 10 mL of acetone and vortexed every 15 min for 2 h. The mixture was then centrifuged at 1500× *g* for 15 min, and the supernatant was collected. Triplicate extractions were performed from each treatment and pooled before analysis.

#### 2.3.1. Quantification of β-Carotene by UV-Vis Spectrophotometry

A stock solution of β-carotene standard (0.3 mg/10 mL acetone) was prepared, and serial dilutions were made to obtain 0.2, 0.5, 2.0, 4.0, 7.5, and 15 µg/mL. Absorbance values were measured at 453 nm by using the UV-Vis spectrophotometer (HACH DR 3900, Hach Company, Düsseldorf, Germany). A calibration curve was drawn between absorbance and concentration to quantify β-carotene in oil samples [14]. The corresponding calibration curve is provided in the Supplementary Information (Figure S1A).

#### 2.3.2. Determination of Extraction Efficiency

The extraction efficiency of β-carotene transfer from *Pouteria campechiana* pulp into coconut oil was calculated to evaluate the performance of the enrichment process. The initial β-carotene concentration in the fresh pulp was determined as described in Section 2.3.1. The efficiency (%) was calculated according to the following equation:

(3) $$Extraction\;efficiency\left(\%\right)=\frac{C_{oil}}{C_{fruit}}\times 100$$

where C_oil_ is the β-carotene concentration (µg/mL) measured in the enriched coconut oil and C_fruit_ is the β-carotene concentration (µg/g of FW) of the *P. campechiana* pulp used for enrichment.

### 2.4. Chemical Composition Analysis of Oils

#### 2.4.1. Saponification Value (SV)

Saponification value was determined according to the method described in AOAC method 920.160 (2019) [15]. Five grams of oil samples were mixed with 4% ethanolic KOH, and the mixture was titrated against 0.5 N HCl using phenolphthalein as the indicator. The saponification value was calculated based on titration readings of the sample and blank.

Saponification Value = (56.11 × (B − S) × N)/W(4)

where

B—Volume in mL of standard HCL required for blank test;
S—Volume in mL of standard HCL required for the sample test;
N—Normality of the standard HCL;
W—Weight of oil (g).

#### 2.4.2. Acid Value (AV)

Acid value was determined according to the ISO 660:2009 (ISO, 2009) [16] method. Ten grams of the oil sample was dissolved in 99% ethanol, gently heated until homogeneous and titrated with 0.1 N KOH using phenolphthalein as the indicator. AV was calculated using the following equation.

Acid Value = (56.11 × Used KOH (mL) × Normality of KOH)/(Sample weight (g))(5)

#### 2.4.3. Peroxide Value (PV)

Peroxide value was measured using the AOAC 965.33 [15] method. Ten grams of the oil sample was mixed with 30 mL of acetic acid-chloroform (3:2) solution, followed by the addition of 1 mL saturated KI and 30 mL of distilled water. The liberated iodine was titrated with 0.01 N Na_2_S_2_O_3_ using 1% starch solution as the indicator. PV was calculated using the following equation.

Peroxide value = (Titrate value (mL) × Normality of Na_2_S_2_O_3_ ×1000)/(Sample weight (g)) (6)

#### 2.4.4. Iodine Value (IV)

Iodine value of the oil samples was determined according to the AOAC 920.159 method [15] with some modifications described by [17]. Approximately 25 mL of chloroform and 25 mL of Wij’s solution were added to the sample in an iodine flask. After 30 min of dark incubation, the mixture was titrated with 0.1 N Na_2_S_2_O_3_ using starch as the indicator. IV was expressed as grams of iodine absorbed by 100 g of oil.

Iodine value = (12.69 × (V_b_ − V_s_) × Normality of Na_2_S_2_O_3_)/(Sample weight (g)) (7)

#### 2.4.5. Color Variation

Color parameters (L*, a*, b*) were determined using a colorimeter (BIOBASE BC-110/200, BIOBASE, Jinan, China) under semi-dark conditions. Hue angle (h°), chroma value (C*), yellowness index, and redness/yellowness ratio were calculated as described by [18,19].

### 2.5. Phytochemical Analysis of Oils

#### 2.5.1. Preparation of Methanolic Extracts

Methanolic extracts of both enriched and commercial coconut oils were prepared following the method described by [20] with slight modifications. One milliliter of oil was mixed with 10 mL of 95% methanol (1:10 *v*/*v*) and vortexed intermittently for 1 h. The mixture was stored in the dark for 24 h, then centrifuged at 2500× *g* for 10 min, and the supernatant was collected for analysis.

#### 2.5.2. Total Phenolic Content (TPC)

Total polyphenol content was determined using Folin-Ciocateu (FC) method [21]. Briefly, 200 µL of methanolic extract was mixed with 1 mL of the FC reagent and after 2 min with 1 mL of 7.5% NaCO_3_ was added. After two 2-min durations, the solution was mixed with 1 mL 7.5% Na_2_CO_3_. The volume was adjusted to 5 mL with distilled water and incubated in the dark for 30 min. The absorbance of the mixture was taken at 765 nm by using a UV-visible spectrophotometer (HACH DR 3900, Hach Company, Düsseldorf, Germany). Results were expressed as mg gallic acid equivalent (GAE) per gram of the oil. The corresponding calibration curve is provided in the Supplementary Information (Figure S1B).

#### 2.5.3. Total Flavonoid Content (TFC)

Total flavonoid content was determined according to the aluminum chloride colorimetric method [22]. Methanolic extract of the sample (1 mL) or the standard solution, or the blank solution was mixed with 4 mL of distilled water and sequentially treated with 0.3 mL of 5% NaNO_2_, 0.3 mL of 10% AlCl_3_, and 2 mL of 1 M NaOH at the specific intervals. The absorbance was measured at 510 nm, and the results were expressed as a mg of quercetin equivalent (QE) per g of oil. The corresponding calibration curve is provided in the Supplementary Information (Figure S1C).

#### 2.5.4. DPPH Radical Scavenging Assay

Antioxidant activity was assessed by using the DPPH radical scavenging assay with slight modifications [23]. One milliliter of 0.1 mmol DPPH solution was added to 0.1 mL of the sample extract or the standard solutions. The mixture was vortexed and incubated in the dark for 30 min, and absorbance was recorded at 517 nm. The antioxidant activity was expressed as mg Trolox equivalent per g (TE mg/g). The corresponding calibration curve is provided in the Supplementary Information (Figure S1D).

### 2.6. Statistical Analysis

Experiment data were analyzed using Design Expert software (version 13.0, Stat-Ease Inc., Minneapolis, MN, USA). Model adequacy was evaluated based on lack of fit, coefficient of determination (R^2^), adjusted R^2^, and coefficient of variation. Response surface plots were also generated using Design Expert software. Comparative analyses of enriched and commercial oils were performed using an independent sample *t*-test in MINITAB 20.0 (Windows). All experiments were conducted in triplicate (n = 3) and data were expressed as mean ± standard deviation.

## 3. Results and Discussion

### 3.1. β-Carotene Content in Canistel

The initial β-carotene concentration in the *Pouteria campechiana* pulp used in this study was 21.29 ± 1.45 µg/g dry weight (DW). This value falls within the range previously reported for Sri Lankan *Pouteria campechiana*, confirming consistency with existing literature. For instance, the study conducted by Lanerolle et al. reported that total carotenoid contents ranged from 1.9 to 23.5 mg/g dry weight (DW) in Sri Lankan *Pouteria campechiana*, with individual β-carotene up to ~156 µg/g DW [24]. These findings collectively confirm that *P. campechiana* is a rich natural source of provitamin A carotenoids, particularly β-carotene, which can be enzymatically converted to retinol in the human body [24]. Thus, its incorporation into edible oils provides a promising dietary strategy to alleviate vitamin A deficiency through food-based fortification.

β-Carotene concentration was determined using UV–Vis spectrophotometry at 453 nm with a β-carotene standard, which provides reliable quantification of this pigment under the applied extraction conditions. However, as *P. campechiana* contains other carotenoids with different spectral characteristics, future work employing HPLC analysis is recommended to confirm compound identity and quantify individual carotenoids.

### 3.2. Model Fitting and Statistical Validation

The response surface model was developed using a Central Composite Design (CCD) to determine the optimal extraction parameters for obtaining maximum β-carotene content from *Pouteria campechiana* into coconut oil. The experimental design matrix and corresponding β-carotene responses are presented in Table 2.

Analysis of variance (ANOVA) indicated that the quadratic polynomial model adequately represented the experimental data (Table 3). The high coefficient of determination (R^2^ = 0.9970) and the non-significant lack of fit (*p* = 0.6815) confirmed the suitability of the fitted model for predicting β-carotene content. The F-value of 9.57 (*p* < 0.05) also demonstrated the overall significance of the model. Both independent variables, such as heating temperature (A) and oil percentage (B), along with their quadratic (A^2^, B^2^) and interactive (AB) effects, were statistically significant (*p* < 0.05).

The regression equation describing the relationship between β-carotene content (BC) and the independent variable in coded form is expressed as:

BC = 2.87 − 1.03A + 0.206B − 0.271AB − 0.871A^2^ + 0.152B^2^ (8)

A higher absolute coefficient value of temperature (A) indicates that it exerts a stronger influence on β-carotene degradation than oil percentage. The negative sign of the temperature coefficient reflects the thermal sensitivity of β-carotene, whereas the positive contribution of oil percentage highlights its protective (reducing oxidative degradation) and solubilizing role in the lipid matrix [25]. Similar trends have been reported for lipid-based carotenoid extractions, where the solvent-to-solid ratio and moderate thermal input significantly affect yield and stability [25,26,27]. The closed agreement between the adjusted R^2^ (0.9970) and predicted R^2^ (0.8788) values further support the model’s predictive reliability.

The optimization focused on heating temperature and oil-to-solid ratio, which preliminary tests identified as the most influential factors affecting carotenoid transfer under solvent-free conditions. Variables such as extraction time and agitation speed were kept constant, as their effects were minor within the selected processing window. Simplifying the model to two dominant variables allowed for better predictive accuracy while maintaining practical relevance for small-scale and industrial applications. Future work should expand this model to include additional parameters (e.g., time, agitation speed) and responses (e.g., color stability and antioxidant activity) to further enhance process optimization.

### 3.3. Effect of Extraction Conditions on β-Carotene Enrichment

**Figure 1:**
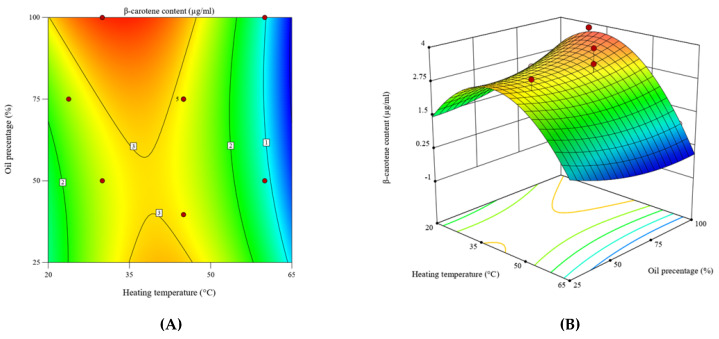
Two-dimensional contour plot illustrating the interactive influence (**A**) and 3D surface plot of the optimized response surface illustrating the interactive influence of heating temperature and oil percentage on β-carotene content (**B**).

The interactive effects of heating temperature and oil percentage on β-carotene incorporation from *Pouteria campechiana* pulp into coconut oil were evaluated using contour and three-dimensional surface plots (Figure 1A,B). The response surface revealed that β-carotene enrichment increased progressively with temperature up to approximately 50 °C and then declined sharply at higher temperatures. Similarly, increasing the oil percentage enhanced β-carotene recovery until about 70–80%, after which further increases produced only marginal improvement, suggesting a saturation plateau.

These patterns indicate that mild heating facilitates diffusion and pigment release, whereas excess heat promotes isomerization and oxidative degradation of β-carotene. Thermal activation softens that plant matrix, decreases viscosity, and improves carotenoid mobility from chromoplasts into the lipid phase [28]. However, β-carotene is thermolabile; above ~55 °C it undergoes structural isomerization to *cis*-forms with reduced provitamin A activity, followed by oxidative cleavage into apocarotenoids [12,29,30].

The oil-to-solid ratio also modulates solubilization and mass transfer kinetics. At low oil fractions, sufficient solvent volume limits pigment diffusion, while very high oil levels dilute the concentration gradient and reduce extraction efficiency. Such behavior has been reported for oil-based extraction of carotenoids from carrot, pumpkin, and mango matrices, where a plateau occurs once solubilization equilibrium is reached [20,31].

**Figure 2:**
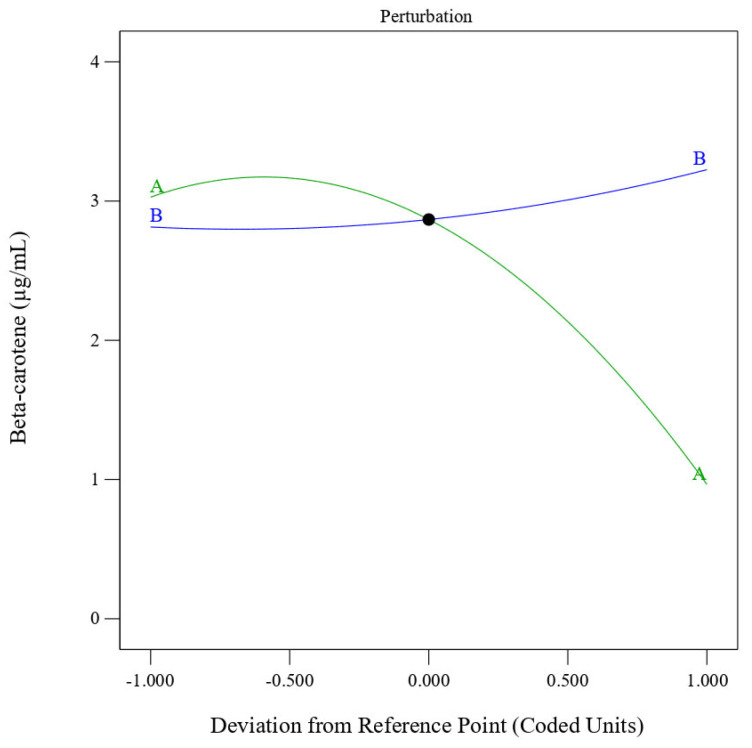
Perturbation graph represents the effect of each independent variable (heating temperature) (A) and oil percentage (B) on β-carotene-enriched coconut oil from *Pouteria campechiana*.

The perturbation plot (Figure 2) further highlights that temperature (Factor A) exerts the strongest effect on β-carotene yield, showing a steep slope, while oil percentage (Factor B) displays a gentle, positive trend. This confirms that controlling thermal input is critical for maximizing carotenoid retention. Comparable results have been reported in ultrasonic and response surface optimized extractions of carotenoids from pumpkin and papaya oils, where moderate heating improved yield, but higher temperatures caused rapid pigment loss [32,33].

Under the optimized conditions determined by the model (52.4 °C and 50% oil), the predicted β-carotene concentration in enriched coconut oil was 2.22 µg/mL, closely matching the experimental value of 2.215 µg/mL (Table 4). The minimal deviation (<0.3%) validates the model’s predictive reliability. It demonstrates that mild heating coupled with an appropriate oil fraction provides a thermodynamically favorable window for carotenoid migration without compromising molecular stability.

Overall, these results confirm that careful control of both temperature and solvent ratio is essential for developing nutrient-fortified oils. The optimized conditions achieved in this study strike a balance between effective extraction kinetics and minimal degradation, yielding a stable, β-carotene-rich coconut oil suitable for functional food applications.

### 3.4. Physicochemical Properties of Enriched Coconut Oil

The physicochemical characteristics of β-carotene-enriched coconut oil and the control (unenriched) oil are presented in Table 5. The enrichment process caused only minor variations in saponification value (SV), acid value (AV), iodine value (IV), and peroxide value (PV), remaining within the acceptable limits specified for edible oils.

The saponification of the enriched oil (248.12 mg KOH/g) was comparable to that of the control (251.23 mg KOH/g), indicating no significant alteration in the triglyceride structure during the heating and enrichment process. A stable SV implies that the molecular integrity of the oil was maintained and no substantial hydrolytic or polymerization reactions occurred which agrees with literature that moderate heating does not affect the ester linkage stability of medium chain triglycerides in coconut oil [34].

The peroxide value of the enriched oil (0.05 meqO_2_/kg) was lower than that of the control (0.09 meqO_2_/kg), demonstrating enhanced oxidative stability. This reduction can be attributed to the antioxidant activity of β-carotene, which acts as a singlet-oxygen quencher and radical scavenger in lipid systems [35]. Similar reductions in PV were observed when β-carotene or other carotenoid-rich extracts were incorporated into vegetable oils, where the pigments inhibited the initiation phase of lipid peroxidation [36]. There is no significant difference between acid value of enriched oil and control sample, suggesting reduced hydrolytic rancidity. The presence of carotenoids and associated phenolic compounds may suppress hydrolytic enzyme activity and secondary oxidation reactions that form free fatty acids, as reported for other natural antioxidant-fortified oils [37]. The iodine value of the enriched oil was marginally lower than that of the control, reflecting minor oxidative consumption of double bonds or limited carotene oxidation during heating. Comparable trends were reported in enriched palm and sunflower oils flowing through antioxidant incorporation and mild thermal treatment [38].

Overall, these results indicate that β-carotene enrichment enhanced the immediate oxidative stability of coconut oil under the tested conditions without inducing adverse chemical alterations. The lower PVs and AVs signify improved resistance to oxidative and hydrolytic degradation, while the consistent SV and small IV variation confirm that the lipid matrix remained structurally stable. The protective effect of β-carotene thus extends beyond its nutritional contribution and improves practical stability during storage and moderate cooking which is an advantage for tropical food systems where oxidative deterioration is a common quality concern. However, this study did not include a storage stability assessment. Future studies should evaluate β-carotene retention and oxidative stability of the enriched oil over time to confirm its shelf-life performance.

### 3.5. Color Characteristics of Enriched Oil

The color parameters of the β-carotene-enriched and control coconut oils are summarized in Table 6.

**Figure 3:**
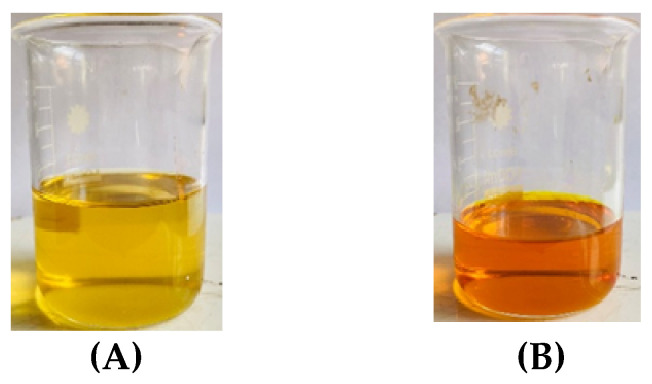
Images of (**A**) commercial coconut oil and (**B**) β-carotene-enriched coconut oil.

Incorporation of *Pouteria campechiana* pulp markedly influenced the color attributes of coconut oil (Figure 3). The enriched oil exhibited a significantly higher b* value (yellowness) and chroma compared with the control, while a slight reduction in lightness was observed. The hue angle shifted toward a more yellow-orange region, reflecting the incorporation of β-carotene pigment.

These findings confirm that color development is a reliable indicator of β-carotene retention in the oil phase. The strong correlation between b* and total carotenoid content has been demonstrated in several studies on carotenoid-enriched edible oils and oleoresins [39,40]. For instance, Meléndez-Martínez showed that increased β-carotene concentration in lipid matrices results in pronounced yellowness and higher chroma values due to its extended conjugated double-bond system, which absorbs in the blue-violet region of the visible spectrum [12].

The enhanced yellowness of the enriched coconut oil not only confirms successful pigment transfer but also provides a natural visual quality marker. Moreover, the color enhancement achieved without synthetic dyes aligns with current consumer preference for clean-label and naturally colored products. The stability of this coloration during mild heating and storage is an important quality attribute as pigment degradation typically precedes sensory deterioration. The results therefore demonstrated that the *Pouteria campechiana* can serve as a promising natural source of β-carotene for color and nutritional enhancement of coconut and other edible lipid systems.

### 3.6. Functional Properties of Enriched Oil

**Figure 4:**
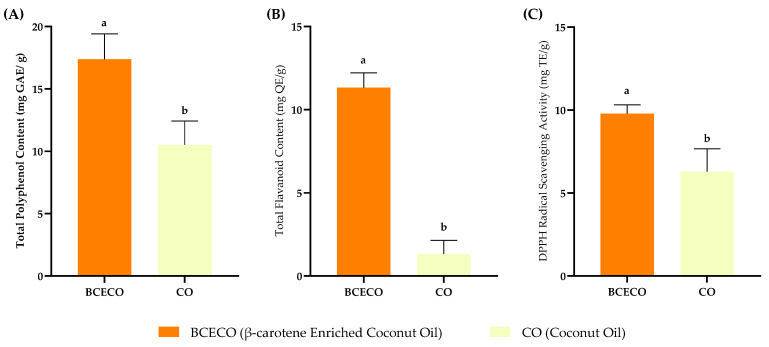
Functional properties of β-carotene-enriched coconut oil and untreated coconut oil. (**A**) Total Polyphenol content, (**B**) Total Flavonoid Content, and (**C**) DPPH radical Scavenging Activity. Values are mean ± standard deviation (n = 3), while different letters in each bar indicate significant differences (*p* ˂ 0.05).

The functional properties of β-carotene-enriched coconut oil and the control oil are presented in Figure 4. Enrichment of coconut oil with *Pouteria campechiana* resulted in significant (*p* < 0.05) increases in measured TPC, TFC, and radical scavenging capacity compared to the control samples.

The enhanced antioxidant potential of the enriched oil can be attributed to the co-extraction of phenolic and flavonoid compounds from the fruit matrix and to the intrinsic antioxidant activity of β-carotene. Mild thermal conditions applied during the RSM-optimized enrichment process likely facilitated the release of bound phenolics and flavonoids by disrupting phenol-protein and phenol-polysaccharide interactions, thereby improving their transfer into the lipid phase [41,42]. β-carotene itself contributes to antioxidant protection through single-oxygen quenching and radical scavenging, acting synergistically with phenolics to enhance overall antioxidant performance [43].

The findings are consistent with previous research, where oil enrichment with carotenoid or phenolic rich plant matrices significantly improved antioxidant properties. For example, flaxseed oil enriched with sea-buckthorn pomace [44] and extra virgin olive oil fortified with tomato-derived lycopene [45]. The present study demonstrates that mild, solvent-free enrichment of coconut oil can effectively transfer both lipophilic carotenoids and co-extractable polar phenolics into the lipid phase, yielding an oil with superior oxidative stability and functional use. Such enhancement offers a sustainable approach to developing value-added oils with improved nutritional and antioxidant attributes.

Furthermore, a preliminary estimation of material costs indicated that the preparation of 1 L of β-carotene–enriched coconut oil required approximately 1 L of refined coconut oil (≈850 LKR/ L) and 2.0 kg of *Pouteria campechiana* pulp (≈400 LKR/ kg), giving a total raw-material cost of about 1250 LKR (≈USD 4.21). Because the process is solvent-free and involves only edible, locally available materials, additional chemical or disposal costs are negligible. This suggests that the proposed method is both cost-effective and environmentally sustainable, aligning with current trends in green processing and local resource valorization.

## 4. Conclusions

This study successfully optimized the solvent-free enrichment of coconut oil with β-carotene extracted from *Pouteria campechiana* using response surface methodology. The fitted quadratic model (R^2^ = 0.9970, *p* < 0.05) reliably predicted β-carotene incorporation, identifying 52.4 °C and a 50% oil-to-solid ratio as the optimal conditions. Under these conditions, experimental and predicted β-carotene concentrations were in close agreement, validating the model’s accuracy. Mild heating combined with an appropriate oil ratio enhanced carotenoid diffusion from the fruit matrix while minimizing thermal degradation, resulting in a stable, β-carotene-rich coconut oil. Enrichment did not adversely affect key physicochemical properties such as saponification, acid, iodine and peroxide values remained within the limits for edible oil while oxidative stability was improved, as evidenced by reduced peroxide and acid values. Color results confirmed significant increase in yellowness (b*) and chroma providing a natural visual quality indicator of carotenoid retention. Moreover, enriched oil exhibited higher total phenolic content, flavonoid content and DPPH radical-scavenging activity than control reflecting synergistic antioxidant effects between co-extracted phenolics and β-carotene. Overall, the process demonstrated in this work offers a green, solvent-free and thermally mild method to produce nutritionally and functionally enhanced coconut oil using locally available carotenoid sources. The β-carotene-enriched oil possesses improved oxidative stability, natural coloration and potential health benefits, making it a promising ingredient for functional foods and nutraceutical applications. Future studies should investigate the long-term storage stability and sensory attributes of the enriched oil to evaluate its consumer acceptability. In addition, the bioaccessibility and intestinal uptake of β-carotene should be examined using in vitro simulated digestion models to validate its nutritional efficacy. Further research could also explore alternative oil matrices rich in unsaturated fatty acids and the development of stable dried or powdered *Pouteria campechiana*–based ingredients for diverse food applications.

## Figures and Tables

**Table 1:** Independent variables and their corresponding levels used for β-carotene enrichment.

Independent Variable	Symbol	Levels				
		−α (−1.141)	−1	0	1	α (1.141)
Temperature (℃)	X_1_	−23.79	30	45	60	66.21
Oil percentage (% oil: solid)	X_2_	−39.64	50	75	100	110.36

**Table 2:** Matrix of experimental design (real values) with response in terms of β-carotene content and extraction efficiency.

Run	Independent Variable		Response Variable	Extraction Efficiency (%)
	Factor 1 Heating Temperature (°C)	Factor 2 Oil Percentage (%)	β-Carotene Content (µg/mL)	
1	30	50	2.5136 ± 0.29	10.52 ± 0.38
2	45	110.35	3.3711 ± 0.25	31.14 ± 1.09
3	60	50	0.6981 ± 0.03	2.92 ± 1.88
4	45	39.64	3.2908 ± 0.31	10.92 ± 0.62
5	60	100	0.9236 ± 0.10	7.73 ± 1.05
6	30	100	3.8222 ± 0.52	31.99 ± 1.76
7	45	75	3.2126 ± 0.52	20.17 ± 0.17
8	45	75	3.7887 ± 0.41	23.78 ± 0.27
9	23.79	75	2.5360 ± 0.23	15.92 ± 0.11
10	66.21	75	0.0326 ± 0.00	0.20 ± 0.03
11	45	75	2.5538 ± 0.64	16.03 ± 0.59
12	45	75	2.5293 ± 0.29	15.88 ± 0.14
13	45	75	2.2546 ± 0.51	14.15 ± 0.53

**Table 3:** ANOVA for β-carotene response.

Source	Sum of Squares	df	Mean Square	F-Value	*p*-Value
Model	14.93	5	2.99	9.57	<0.0049 ^a^
A-Temperature	8.52	1	8.52	27.31	<0.0012 ^a^
B-Oil percentage	2.40	1	0.3393	7.69	<0.0370 ^a^
AB	1.75	1	0.2933	5.61	<0.0410 ^a^
A^2^	5.28	1	5.28	5.23	<0.0245 ^a^
B^2^	0.63	1	0.1607	2.02	<0.0410 ^a^
Residual	2.18	7	0.3119		
Lack of Fit	0.6270	3	0.2090	0.5372	0.6815 ^b^
Pure Error	1.56	4	0.3890		
Cor Total	17.11	12			

^a^ Significant; ^b^ Not significant, R^2^ = 0.9970, Adjusted R^2^ = 0.9912, Predicted R^2^ = 0.8788.

**Table 4:** Validation of the mathematical model.

Dependent Variable	Experimented Value	Predicted Value
β-carotene content	2.215 (µg/mL)	2.22 (µg/mL)

**Table 5:** Chemical properties of β-carotene-enriched coconut oil and commercially available coconut oil.

Test Parameter	Type of Oil	
	β-Carotene-Enriched Coconut Oil	Coconut Oil
Saponification value (mg KOH/g)	248.12 ± 1.2 ^a^	251.23 ± 2.3 ^a^
Peroxide value (meqO_2_/kg)	0.05 ± 0.01 ^b^	0.09 ± 0.01 ^a^
Acid value (mg KOH/g)	0.10 ± 0.01 ^a^	0.17 ± 0.02 ^a^
Iodine Value (I_2_ g/100 g)	6.88 ± 0.12 ^b^	7.91 ± 0.10 ^a^

Values are mean ± standard deviation (n = 3), while different letters for values in each row indicate significant differences (*p* ˂ 0.05).

**Table 6:** Color parameters of β-carotene-enriched coconut oil and untreated coconut oil.

Color Parameter	β-Carotene-Enriched Coconut Oil	Coconut Oil
L*	30.09 ± 0.51 ^a^	30.12 ± 0.68 ^a^
a*	5.31 ± 0.32 ^b^	6.29 ± 0.71 ^a^
b*	8.67 ± 0.33 ^a^	1.75 ± 0.17 ^b^
Hue angle (h°)	58.51 ± 1.82 ^a^	15.55 ± 2.20 ^b^
Yellowness index	41.16 ± 1.72 ^a^	8.30 ± 0.83 ^b^
Redness/yellowness ratio	0.61 ± 0.04 ^b^	3.59 ± 0.54 ^a^
Chroma (C*)	10.17 ± 0.33 ^a^	6.53 ± 0.69 ^b^

Values are mean ± standard deviation (n = 3), while different letters for values in each row indicate significant differences (*p* ˂ 0.05).

## Data Availability

The original contributions presented in this study are included in the article/Supplementary Material. Further inquiries can be directed to the corresponding author.

## Supplementary Materials

The following supporting information can be downloaded at: https://www.mdpi.com/article/10.3390/foods14223947/s1, Figure S1. Calibration curves for (A) β-carotene standard used to determine β-carotene content, (B) gallic acid standard used to determine total polyphenol content, (C) quercetin standard used to determine total flavonoid content, and (D) Trolox standard used to determine DPPH radical scavenging activity.

## Abbreviations

ANOVA	Analysis of Variance
AV	Acid Value
β-Carotene	Beta-Carotene
CCD	Central Composite Design
DPPH	2,2-Diphenyl-1-picrylhydrazyl
GAE	Gallic Acid Equivalent
IV	Iodine Value
L*, a*, b*	CIE Color Space Coordinates (Lightness, Red-Green, Yellow-Blue)
PV	Peroxide Value
QE	Quercetin Equivalent
R^2^	Coefficient of Determination
RSM	Response Surface Methodology
SV	Saponification Value
TFC	Total Flavonoid Content
TPC	Total Phenolic Content
TE	Trolox Equivalent
UV–Vis	Ultraviolet–Visible Spectrophotometry
