# Supplementary material for: Optimization of β-Carotene Enrichment of Coconut Oil from Canistel (Pouteria campechiana L.) Using Response Surface Methodology
Source: Foods. 2025 Nov 18;14(22):3947. doi: 10.3390/foods14223947 (PMC12651562; doi:10.3390/foods14223947)
Supplement: Supplementary file 1 [file foods-14-03947-s001.zip › foods-3978153-supplementary.pdf]

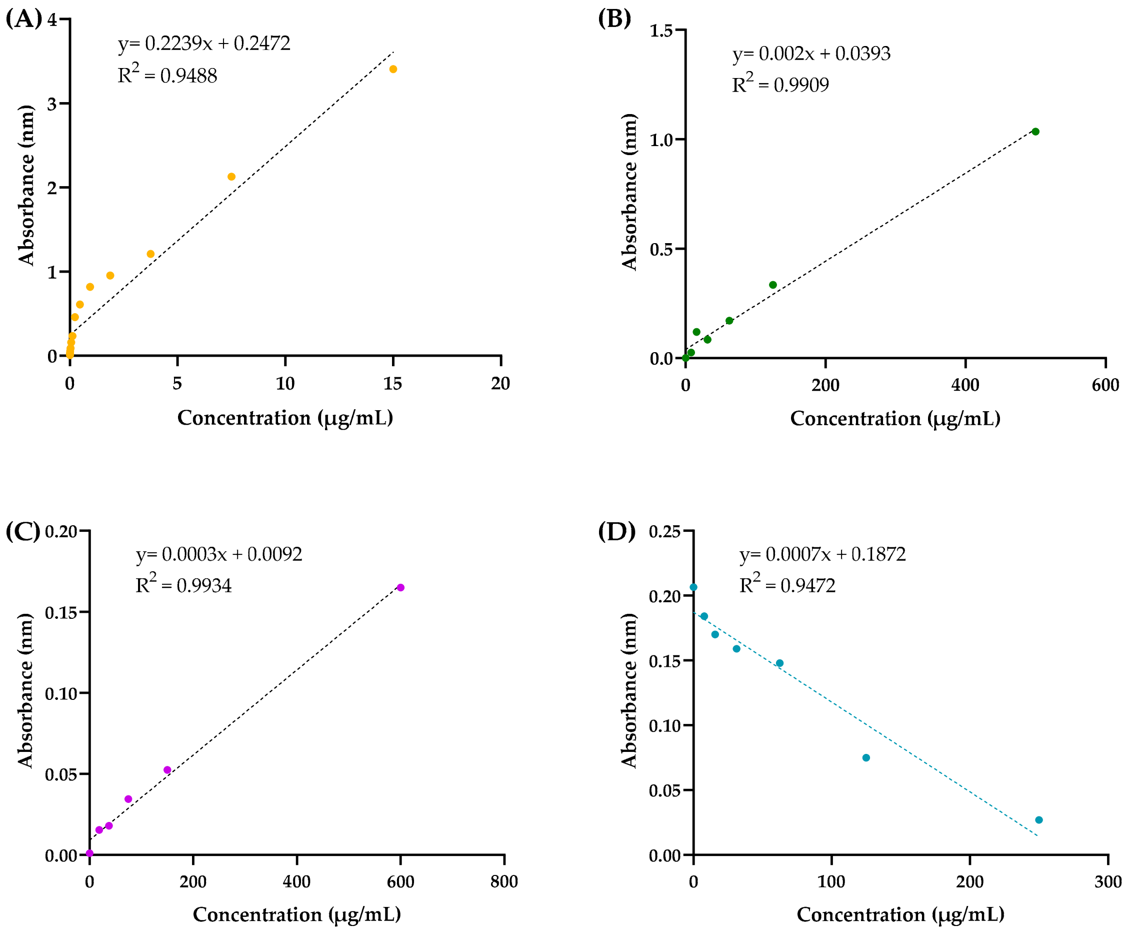

Figure S1: Calibration curves for (A) β-carotene standard used to determine β-carotene content, (B) gallic acid standard used to determine total polyphenol content, (C) quercetin standard used to determine total flavonoid content, and (D) Trolox standard used to determine DPPH radical scavenging activity.
